# Preliminary Evaluation of a Hybrid Scan Transfer Workflow for Complex Full-Arch Implant Rehabilitations

**DOI:** 10.3390/dj14060358

**Published:** 2026-06-10

**Authors:** Roberto Scrascia, Maria Eleonora Bizzoca, Lino Locurcio, Irene Catalano, Francesco Antonucci, Annika Barone, Enrico Cataneo, Gabriele Cervino

**Affiliations:** 1 Independent Researcher, 74121 Taranto, Italy; roberto.scrascia@gmail.com; 2 Department of Clinical and Experimental Medicine, University of Foggia, Via Luigi Rovelli 50, 71122 Foggia, Italy; mariaeleonora.bizzoca@unifg.it (M.E.B.); lino.locurcio@unifg.it (L.L.); irene_catalano.555156@unifg.it (I.C.); 3 Odontostomatology Unit, “S. G. Moscati” Hospital, ASL Caserta, Via Gramsci 1, 81031 Aversa, Italy; francescoantonucci@libero.it; 4 Independent Researcher, 80125 Naples, Italy; annikabarone@gmail.com; 5 Department of Health Sciences, School of Dentistry, Magna Graecia University of Catanzaro, Viale Europa, 88100 Catanzaro, Italy; enrico.cataneo@unicz.it; 6 Department of Biomedical and Dental Sciences, Morphological and Functional Images, University of Messina, G. Martino Polyclinic, 98125 Messina, Italy

**Keywords:** digital dentistry, intraoral scanner, scan bodies, full-arch rehabilitation, implant prosthodontics, hybrid workflow

## Abstract

**Background/Objectives**: Digital workflows have significantly improved implant prosthodontic procedures; however, full-arch and complex rehabilitations still present challenges for intraoral scanning because of cumulative stitching errors, limited anatomical landmarks, and reduced scan body stability. **Methods**: This article describes a single proof-of-concept clinical case in which a hybrid Scan Transfer workflow was applied to support implant position transfer during intraoral scanning through stabilization, verification, and supplementary extraoral acquisition steps. The proposed workflow was compared descriptively with a conventional scan body approach in one full-arch implant-supported rehabilitation. Digital datasets were acquired with an intraoral scanner and analyzed by curve-based superimposition to explore relative discrepancies between the two transfer methods. **Results**: In this individual case, the hybrid workflow showed descriptive findings suggestive of lower deviations, particularly in posterior regions, and favorable framework adaptation on the reference model. **Conclusions**: Because the evidence is limited to a single clinical case and no inferential statistical analysis was performed, these findings should be interpreted as preliminary and hypothesis-generating only. Further controlled in vitro and clinical studies with larger samples are required before conclusions can be drawn regarding accuracy, reliability, or clinical superiority.

## 1. Introduction

In recent years, digital technologies have significantly transformed clinical and laboratory workflows in implant dentistry. The adoption of intraoral scanners (IOS) and CAD/CAM systems has enabled the transition from conventional analogue impressions to digital protocols, improving efficiency and facilitating prosthetic and surgical planning [1,2].

Nevertheless, despite their widespread use, fully digital workflows remain challenging in complex rehabilitations, particularly in full-arch and edentulous implant-supported cases.

In these scenarios, the accuracy of digital impressions is influenced by multiple factors, including scanner performance, scanning strategy, operator technique, environmental conditions, and scan body geometry [3,4].

As essential geometric references, scan bodies facilitate the transfer of the implant’s three-dimensional (3D) position to planning software. To minimize alignment errors with their digital counterparts, these components must feature high-contrast geometric markers, flat surfaces for orientation, and opaque materials to prevent light scattering during intraoral capture. Furthermore, the scan body’s height and stability are critical to ensuring full visualization above the soft tissue without impeding scanner access within the oral cavity [5].

While IOS performance is often attributed to software algorithms, recent evidence suggests that the physical geometry of the scan body itself is a primary determinant of trueness. Specifically, shorter, simplified designs (5 mm) have been shown to significantly reduce mean deviations compared to standard 10 mm designs across various clinical configurations [6].

The absence of stable anatomical landmarks in edentulous arches, together with vertical discrepancies among implant scan bodies, may compromise image stitching and data registration, leading to cumulative deviations and reduced trueness, especially in posterior regions [7,8].

Recent in vivo evidence suggests that employing a ‘scan aid’ or optical bridge can significantly mitigate these challenges by providing a continuous geometric reference, particularly for scanners utilizing active triangulation technology [9].

Scan bodies play a critical role in the digital implant workflow by transferring implant position and angulation into the virtual environment. Variations in their design, material, and height have been shown to affect scanning accuracy and alignment during CAD processing, although no consensus exists regarding optimal configurations [10,11]. While IOS offer several practical advantages over conventional impressions, full-arch digital acquisitions remain susceptible to distortion, and the lack of standardized evaluation protocols limits definitive conclusions regarding their reliability [12,13]. To address these limitations, hybrid approaches combining digital acquisition with stabilization and verification steps have been proposed. The Scan Transfer system was developed with the aim of improving implant position transfer during intraoral scanning by integrating physical stabilization of scan bodies, scan validation, and supplementary extraoral acquisition steps within a unified workflow.

The aim of this article is to describe the application of the Scan Transfer system in a complex full-arch implant-supported rehabilitation and to provide a preliminary, descriptive comparison of implant position capture obtained with this hybrid protocol and with a conventional scan body approach. Given the single-case design, the study was not intended to establish clinical superiority or generate statistically validated accuracy data, but rather to report the workflow, its technical feasibility, and the observed discrepancies under the specific conditions of this case.

## 2. Materials and Methods

This study reports the application of a newly developed implant scanning protocol (Scan Transfer system) in one complex full-arch rehabilitation and compares it descriptively with a conventional scan body approach. All digital acquisitions were performed using an intraoral scanner within a complete CAD/CAM workflow, including data processing, curve conversion, and superimposition analysis to explore discrepancies in implant position transfer. The methodology included the fabrication of auxiliary components, such as a stabilizing PMMA splint, and the production of a master gypsum model for verification of prosthetic framework adaptation. The clinical protocol was applied in a fully edentulous case treated with an All-on-4 implant configuration. Because only one clinical case was analyzed, all findings are descriptive and exploratory and were not subjected to inferential statistical testing.

### 2.1. Medit i90027 IOS

The Medit i900 intraoral scanner (Medit, Seoul, Republic of Korea) was used for all digital acquisitions performed in this study. The device was employed within a fully digital CAD/CAM workflow to obtain intraoral scans for implant position transfer, provisional prosthesis morphology, occlusal records, and supplementary extraoral acquisitions when required. The scanner supports the export of standard digital file formats, including STL, OBJ, and PLY, allowing integration with prosthetic CAD software for subsequent data processing and framework design. In the present workflow, the acquired datasets were imported into exocad for comparative analysis of the two implant position transfer methods investigated, namely conventional Globalwin scan bodies and the Scan Transfer system. Recent evidence has reported high trueness and precision for full-arch implant scanning with the Medit i900 system [14]. On this basis, the scanner was considered suitable for evaluating the proposed hybrid workflow under the conditions of the present technical investigation. In combination with the stabilizing PMMA splint adopted in the CSS strategy, the device enabled acquisition of the scan body positions and supported the comparative assessment of implant position transfer between the two workflows.

### 2.2. CAD Software–Exocad DentalCAD

Exocad DentalCAD (exocad GmbH, Darmstadt, Germany) was used for digital prosthetic planning, dataset management, and descriptive comparison within the CAD/CAM workflow adopted in this study. STL datasets were imported without additional mesh smoothing, decimation, or manual deformation. The conventional Globalwin and Scan Transfer datasets were oriented using the implant connection/platform region as the primary reference landmark. Curve-based alignment and digital superimposition were then performed to compare the spatial arrangement of the four implant-related reference curves. A central weighted best-fit procedure was used to reduce the influence of a single scan body on the overall alignment and to evaluate the cumulative behavior of the full-arch configuration. The same alignment strategy, software environment, and operator sequence were used for both workflows. Colour maps were used only as qualitative visual aids, whereas inter-curve distances were recorded descriptively in micrometres. No predefined clinical pass/fail tolerance threshold and no inferential statistical analysis were applied; therefore, the superimposition output should be interpreted as a technical descriptive comparison rather than as a validated accuracy test.

### 2.3. Scan Transfer and Globalwin SB

The Scan Transfer scan bodies incorporate a truncated-cone geometry intended to improve stability during intraoral scanning and facilitate accurate implant position transfer. Their design includes asymmetric orientation notches that provide a unique reference for alignment, while the retentive morphology allows splinting between adjacent scan bodies in order to increase rigidity during acquisition and potentially improve scan repeatability. The associated digital library includes multiple heights and configurations for both straight and angulated implants, thereby supporting use in different prosthetic and anatomical conditions. In addition, compatibility with both additive and subtractive manufacturing workflows facilitates integration of the system within contemporary CAD/CAM protocols. In the present study, the Scan Transfer scan bodies were connected directly to the implants according to the manufacturer’s instructions and stabilized with a polymethyl methacrylate (PMMA) splint during the scanning procedure. Digital impressions were then acquired using the Medit i900 intraoral scanner, and the resulting datasets were analyzed in comparison with those obtained using the conventional Globalwin scan body system. Under the conditions of this technical investigation, the Scan Transfer workflow was adopted not only for implant position recording, but also for the subsequent design and milling of the titanium prosthetic frameworks, allowing assessment of its applicability throughout the digital workflow.

Globalwin scan bodies are cylindrical components available with either hexagonal or conical connections, selected according to the implant platform and specific clinical requirements. They are manufactured from medical-grade polyetheretherketone (PEEK; Tecapeek Classix white), a material widely used in implant prosthodontics because of its dimensional stability, biocompatibility, and optical behavior during scanning procedures. In the present study, the evaluated Globalwin scan bodies included the ICD model with an internal hexagonal connection (diameter 3.75 mm; PSC screw included) and the ICDPRA model for Performing Abutment with a conical connection (diameter 4.80 mm; PRAPSCS screw included). The use of PEEK for scan body fabrication has been supported by recent evidence indicating that scan body material may influence the trueness and precision of digital implant impressions [15]. For this reason, the Globalwin components were considered an appropriate conventional reference for the descriptive comparison performed in this case. Although the Globalwin scan bodies were suitable for routine implant position transfer, their cylindrical geometry made splinting less straightforward than with the Scan Transfer system in this specific full-arch application. This observation should not be interpreted as a general limitation of the Globalwin system, but as a case-specific technical consideration related to the workflow tested here.

### 2.4. Moon Night S Xl 3D Printer

In this study, the Moon Night S XL 3D printer was used to fabricate the polymethyl methacrylate (PMMA) splint employed in the CSS strategy, which was designed to stabilize and splint the scan bodies during intraoral scanning. The STL file of the splint was transferred from the dental laboratory to the clinical setting, allowing rapid integration of the manufacturing phase within the digital workflow. The printed splint was produced as an auxiliary component of the hybrid protocol and was intended to provide adequate dimensional consistency for clinical use. Its fabrication represented a relevant step in the workflow, since proper adaptation of the splint to the scan bodies was necessary to improve rigidity during acquisition and to support more consistent implant position transfer under the tested conditions.

### 2.5. PMMA Splint

The splint was fabricated by high-precision three-dimensional printing and produced for immediate clinical application within the digital workflow. Polymethyl methacrylate (PMMA) was selected because of its mechanical stability, fracture resistance, dimensional consistency, and suitability for intraoral use. The splint was designed to fit the Scan Transfer components and to connect adjacent scan bodies, thereby increasing rigidity during acquisition and supporting a more reproducible implant-position record in this specific full-arch case.

Before clinical use, the splint was seated on the Scan Transfer components and checked visually and manually for complete adaptation, absence of rocking, and passive fit. When the device was correctly positioned, it was used as part of the CSS strategy to stabilize the scan bodies during intraoral scanning. Three-dimensionally printed PMMA has been reported to provide satisfactory dimensional accuracy for dental applications and represents a practical digital alternative to conventional resin-based procedures [16]. In this context, its use allowed rapid fabrication of a patient-specific auxiliary device that could be integrated into the scanning protocol while maintaining the dimensional consistency required for subsequent digital and prosthetic phases.

### 2.6. Post-Print Cleaner–Moon Wash 2 (Vertysystem)

The Moon Wash 230 (VertySystem) was used for the post-processing cleaning of the 3D-printed PMMA splint after fabrication. This step was necessary to remove residual non-polymerised resin from the printed surface prior to clinical use and before the subsequent photopolymerisation phase. In the present workflow, the splint was subjected to a medium-intensity cleaning cycle. This post-print processing step was included to obtain a cleaner and more dimensionally consistent auxiliary component, thereby supporting proper fit of the splint on the scan bodies and reliable integration of the device within the hybrid scanning protocol.

### 2.7. Photopolymeriser–Moon Light 3 (Vertysystem)

The Moon Light 331 (VertySystem) was used for the post-curing of the 3D-printed PMMA splint fabricated for the hybrid scanning protocol. This step was carried out after the cleaning phase in order to complete polymerisation of the printed material before clinical use. Within the present workflow, post-curing was performed to support dimensional consistency, structural stability, and appropriate handling of the splint during the subsequent clinical phase. Since correct adaptation of the stabilizing device to the scan bodies was essential for the hybrid protocol, the photopolymerisation step represented a relevant part of the manufacturing process of the auxiliary component employed in this study.

### 2.8. Marmorock 20 Dental Stone (Sialdent)

**Figure 1:**
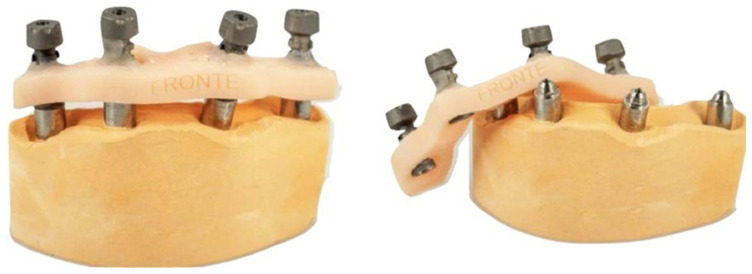
Marmorock 20 dental stone models.

Marmorock 20 (Sialdent) is an extra-hard Type IV dental stone used for applications requiring high precision and mechanical strength (Figure 1). In this study, it was selected to fabricate the master model for validation of the intraoral scanning and milling workflow. Its high compressive strength (60–90 MPa) supports durability during prosthetic framework adaptation, while low linear expansion contributes to dimensional stability. In addition, its thixotropic behavior facilitates accurate pouring with reduced air inclusions and smooth surface reproduction. Master models were therefore produced to assess the trueness of intraoral scans obtained using both the Scan Transfer and Globalwin scan body systems.

### 2.9. Bio Ti Grade 5 Titanium Discs

Grade 5 titanium is a biphasic alloy composed of approximately 90% titanium, 6% aluminum and 4% vanadium. This composition provides the alloy with a minimum tensile strength of 950 MPa, a yield strength of at least 880 MPa, and a minimum elongation of 10%, making it particularly suitable for applications requiring high mechanical performance. Grade 5 titanium is also characterized by high biocompatibility and low density, which reduces the load on implants and surrounding tissues. Grade 5 titanium discs are engineered for CAD/CAM milling, allowing the fabrication of customized prostheses with precise margins and detailed geometry. Their excellent machinability supports the production of complex frameworks with improved implant fit and minimal need for manual refinement. In this study, the discs were used to mill the titanium prosthetic structures with the metal milling unit.

### 2.10. My Evolution Eplus Milling Machine (Santa Barbara)

The My Evolution Eplus32 milling machine (Santa Barbara Dental) is designed for the fabrication of implant-supported prosthetic frameworks, offering high precision and excellent surface adaptation. It supports customisable milling workflows and can process multiple prostheses in a single cycle across various materials, including titanium, cobalt–chromium, PMMA, lithium disilicate, PEEK, resins and composites. Its simultaneous five-axis numerical control enables complex movements and micrometric tolerances suitable for intricate geometries. In this study, all prostheses were milled under identical conditions to minimize variability, and the machine’s repeatability ensured consistent dimensional accuracy, strengthening the comparison between the Scan Transfer and Globalwin implant position transfer method.

## 3. Clinical Case

**Figure 2:**
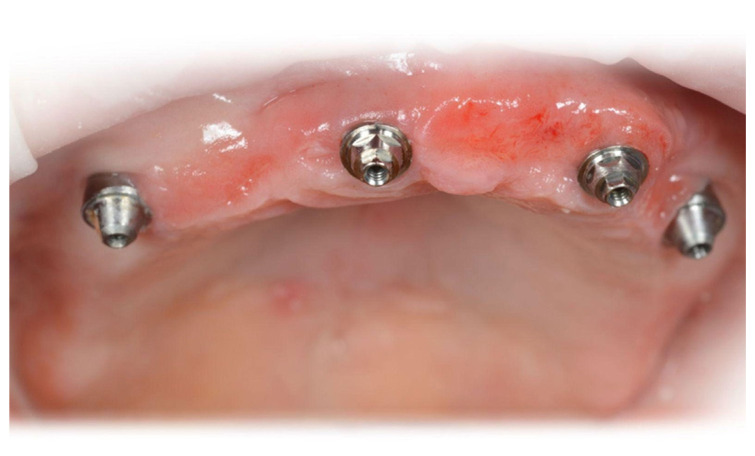
Our clinical case with 4 GlobalWin implants, inserted with the All on 4 technique.

A 71-year-old female patient, non-smoker and without relevant systemic conditions, had previously undergone guided surgery for the placement of four GlobalWin (Biosafin/Umbra) implants using the All-on-4 protocol through a surgical guide (Figure 2). Multi-Unit Abutments (MUAs) were fitted at the time of immediate loading: 17° MUAs on implants in regions 15 and 25, and 0° MUAs on implants in regions 12 and 22.

At the six-month review, the patient returned with the provisional prosthesis delivered at immediate loading. This was clinically useful because the validated provisional provided stable prosthetic references for the subsequent workflow, with vertical dimension, tooth position, lip support, phonetic references, and occlusal relationships already established. These references facilitated the cross-mounting of digital datasets and reduced the need to reconstruct prosthetic parameters from the beginning.

**Figure 3:**
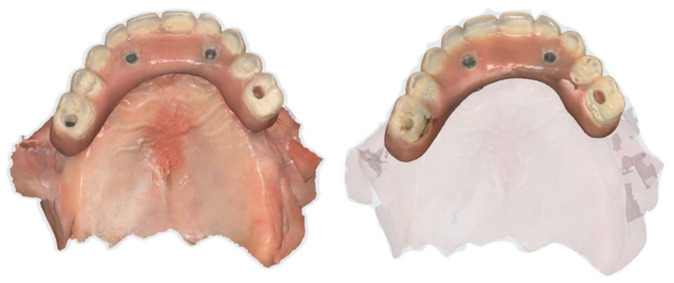
Intraoral and extraoral scan with Medit i900 scanner of the temporary prosthesis resulting from immediate loading.

**Figure 4:**
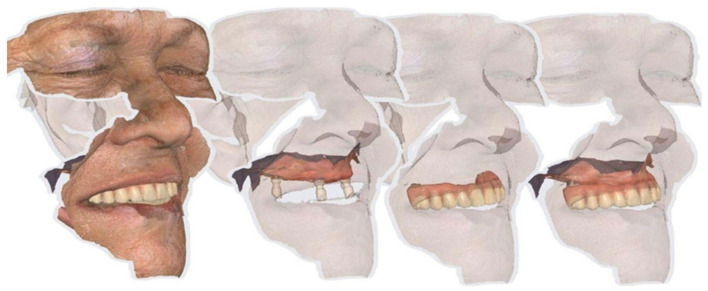
Face scan with Medit i900 scanner.

If a validated provisional restoration had not been available, the workflow would have required additional preliminary records, such as a diagnostic tooth setup, conventional or digital jaw-relation records, an occlusal rim or verification prosthesis, and a separate validation of vertical dimension and tooth position before definitive framework design. In such a scenario, the implant-position transfer protocol could still be applied, but the prosthetic phase would likely be longer and more dependent on additional clinical verification steps. Therefore, the presence of the provisional restoration should be considered a favorable case-specific condition and a limitation when extrapolating the present findings to other full-arch rehabilitations. The provisional prosthesis was scanned both intraorally and extraorally using the Medit i900 scanner (Figure 3), and a face scan was also acquired to support prosthetic integration within the digital workflow (Figure 4).

Following intraoral scanning using the CSS Strategy, an additional extraoral scan of the Scan Transfer–splint assembly was acquired according to the Re-Scan protocol. This supplementary dataset aimed to improve acquisition reliability by reducing potential errors related to patient movement and supporting accurate transfer of implant positions and arch morphology into the CAD software.

**Figure 5:**
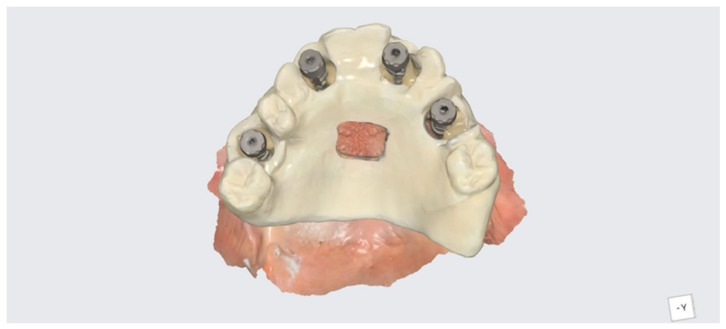
Intraoral scan of the provisional prosthesis prototype connected to Scan Transfer scan bodies, acquired with the Medit i900.

A duplicate of the provisional prosthesis, generated from the previous CAD design (Moon Light, VertySystem, Umbra), was modified by enlarging the implant access openings to accommodate the Scan Transfer components, which were stabilized using the same flowable composite (Figure 5).

This approach enabled efficient intraoral acquisition of implant positions, tooth morphology, and vertical dimension within a single workflow, facilitating accurate cross-mounting with the laboratory. The same protocol was applied to the printed prototype using the manufacturer’s GlobalWin scan bodies; however, their geometry made splinting more challenging.

The CSS Strategy enabled acquisition of a continuous intraoral scan with minimal mesh artefacts. The subsequent extraoral Re-Scan of the splint–Scan Transfer assembly supported dataset integration and correction of potential discrepancies, improving the transfer of implant positions and occlusal relationships. In the final phase, the De-Bug protocol was used to generate a plaster cast from the rigidly splinted scan body arch, producing a master model for confirmation of implant positioning and verification of prosthetic structures prior to the next clinical appointment. Overall, the combined application of the three Scan Transfer protocols appeared to support a clinically manageable workflow under the specific conditions of this case and allowed fabrication of the definitive prosthesis. The final restoration was manufactured using CAD/CAM (computer-aided design/computer-aided manufacturing) with VertySystem fibre and composite materials.

## 4. Results

**Figure 6:**
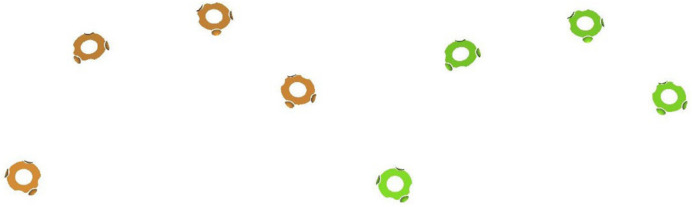
The Scan Transfer curve is shown in orange and the Globalwin scan curve in green. The colour differences indicate that the two curves are not perfectly superimposed.

To enable a descriptive technical comparison of implant-position records obtained with the two scan-body systems (Scan Transfer and Globalwin), all datasets were converted into curves of identical dimensionality and subsequently superimposed to explore spatial discrepancies. As two different scan-body designs were acquired intraorally, a single nominal reference curve was required; for the Scan Transfer units, this curve was provided by the manufacturer, and each component was preliminarily adapted in exocad and aligned using a central weighted best-fit procedure to ensure consistent correspondence across all Scan Transfer scan bodies. For the Globalwin scan bodies, whose geometry differs from Scan Transfer, direct matching to the same nominal curve was not feasible (Figure 6); therefore, conversion was performed by aligning the *Z*-axis value of the Globalwin nominal curve with that of the Scan Transfer curve using the implant connection as the reference landmark. These procedures were used to describe relative discrepancies in this case and were not intended to provide statistically validated accuracy measurements.

**Figure 7:**
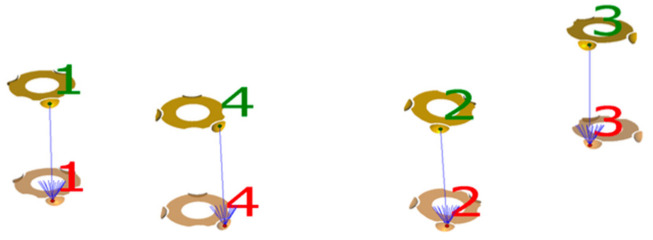
1-2-3-4 are the reference points used to guide the software in aligning the entire curve.

**Figure 8:**
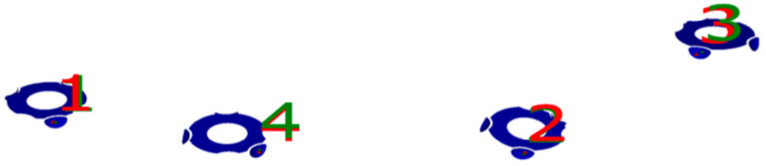
Descriptive colour-map visualization of the curve superimposition. Numbers 1–4 indicate the four reference scan body positions used for curve alignment and descriptive inter-curve distance assessment. Darker blue areas indicate closer local matching according to the software colour scale used for visual inspection; however, because the colour intensity differences are subtle, the figure should be interpreted together with the reported descriptive inter-curve measurements rather than as a stand-alone quantitative result.

Composite curves representing the spatial position of the four scan bodies were superimposed using a central weighted averaging algorithm, avoiding the trivial perfect overlap that would occur if a scan body were matched only to its own nominal geometry and instead capturing the cumulative positional behavior of all four components. After superimposition, colour-differentiated meshes provided an initial qualitative overview, followed by descriptive multi-point curve sectioning and measurement of inter-curve distances. In this case, minimal variation was observed for the first two scan bodies (30 micrometres and 31 micrometres), whereas discrepancies increased from the third unit onwards, reaching a maximum observed deviation of 82 micrometres at the fourth, most posterior position (Figure 7 and Figure 8). These values should be interpreted as descriptive observations from a single case rather than as statistically generalizable evidence of higher accuracy.

**Figure 9:**
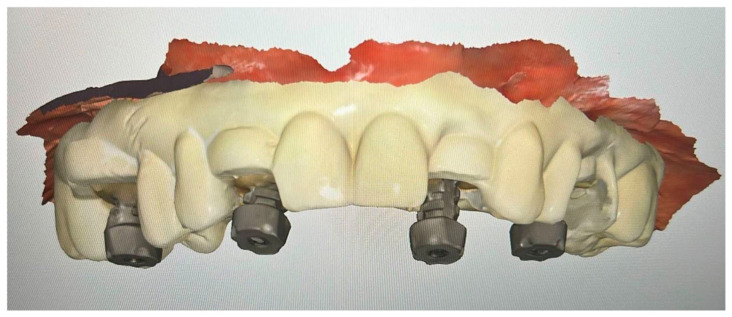
Scan Transfer locked with a copy of the diagnostic prosthesis.

**Figure 10:**
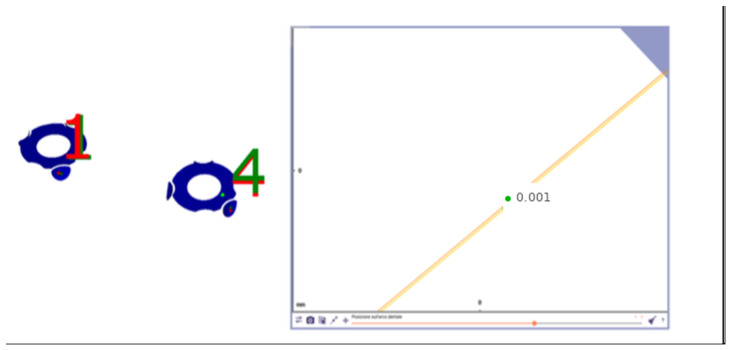
Graphical representation of the alignment, showing negligible discrepancy relative to the nominal reference under the tested conditions. The numerical value indicates the software-generated local alignment discrepancy.

A final internal verification step involved acquiring an additional Scan Transfer scan using a stabilization jig fabricated from the same diagnostic prosthesis (Figure 9). The resulting discrepancy was minimal according to the software-based alignment output, suggesting consistent matching with the nominal reference under the tested conditions (Figure 10). This step was used as an internal workflow check and not as an independent statistical validation.

To assess the clinical relevance of the digital discrepancies, titanium frameworks milled from the Scan Transfer and Globalwin datasets were evaluated on the master gypsum model and intraorally using the same clinical sequence. Framework adaptation on the reference model was assessed by passive seating, visual inspection under magnification, tactile exploration of the implant-framework interface, and a one-screw test to detect rocking or displacement. Intraoral verification was performed by seating each framework on the multi-unit abutments, tightening one terminal screw first, checking for lifting at the opposite side, and then progressively seating the remaining screws while evaluating resistance-free adaptation and clinical stability. In this single case, the Scan Transfer-derived framework appeared to show favorable passive adaptation on the reference model during the clinical and laboratory checks performed. However, these observations remain descriptive and should not be interpreted as evidence of superior clinical performance. These observations were descriptive and were not supported by strain-gauge analysis, radiographic gap measurement, or statistical testing.

## 5. Discussion

The descriptive comparison between the Scan Transfer system and the Globalwin scan bodies showed case-specific differences in implant-position transfer, with the most pronounced discrepancies observed in the posterior segments of the arch. Under the conditions of the present single-case technical investigation, the hybrid Scan Transfer workflow showed descriptive findings suggestive of consistent positional transfer; however, this observation should be interpreted cautiously and cannot be generalized without larger controlled studies.

Superimposition of the digital curves showed that discrepancies between the two workflows remained limited in the anterior region (approximately 30–31 micrometres), whereas larger deviations were observed in the posterior area, reaching 67–82 micrometres for the conventional scan body workflow in this case. Because no repeated measurements across multiple patients or operators were performed, these findings should be regarded as descriptive and hypothesis-generating. They suggest that stabilization and verification steps may contribute to transfer consistency, but they do not prove a statistically validated improvement in accuracy.

The implementation of a stabilized hybrid protocol is consistent with previous evidence indicating that auxiliary scan aids can reduce linear deviation across edentulous spans [9]. The present case supports the technical rationale for providing geometric continuity and scan body stabilization during full-arch acquisition, although the single-case design prevents any definitive conclusion regarding the magnitude or reproducibility of this potential benefit.

The increased discrepancies observed in the posterior measurements are also consistent with systematic reviews reporting that digital accuracy tends to decrease as implant angulation increases and as the edentulous span becomes longer [17]. From this perspective, the present results further support the rationale for incorporating stabilization strategies in complex scanning protocols, particularly in situations where conventional intraoral scanning may be more vulnerable to stitching errors and reduced alignment reliability.

Overall, the present results support the feasibility of the Scan Transfer workflow as a possible approach for managing implant-position transfer in complex rehabilitations. However, the findings remain preliminary because they derive from one proof-of-concept clinical application under controlled conditions. Further in vitro and clinical studies involving larger and more heterogeneous samples, repeated scans, multiple operators, independent reference datasets, and predefined statistical analyses are required to confirm reproducibility, accuracy, and broader applicability.

## 6. Conclusions

Within the limitations of this single-case technical report, the Scan Transfer workflow was feasible and showed promising descriptive behavior under complex clinical conditions, particularly in the posterior regions of the arch, where implant-position transfer is generally more challenging. In the present proof-of-concept application, the hybrid approach showed descriptive findings that may suggest lower observed discrepancies and favorable framework adaptation under the specific conditions tested; however, these observations must be interpreted cautiously because they are based on one patient and were not statistically validated. By integrating stabilization, verification, and supplementary extraoral acquisition steps, the proposed protocol may represent a useful hybrid strategy for selected full-arch implant rehabilitations. Nevertheless, the present data do not allow definitive conclusions regarding improved accuracy, reliability, or clinical superiority. Further controlled in vitro and clinical investigations are needed to determine whether the observed findings can be reproduced across broader and more heterogeneous clinical settings.

## Data Availability

The data supporting the findings of this technical report are available from the corresponding author upon reasonable request. The data are not publicly available because they include clinical information related to a single patient and are subject to privacy restrictions.
